# Association of sugar-sweetened beverage consumption and cardiorespiratory fitness with executive function: a cross-sectional survey based on Tajik adolescents at high altitude in China

**DOI:** 10.3389/fpubh.2025.1554136

**Published:** 2025-03-03

**Authors:** Lingyun Sun, Tianqing Xue, Zhimin Zhao

**Affiliations:** ^1^School of Sports and Health Management, Henan Finance University, Zhengzhou, China; ^2^School of Physical Education, Chizhou University, Chizhou, China; ^3^School of Physical Education and Sports, Shihezi University, Shihezi, China

**Keywords:** SSB, cardiorespiratory fitness, executive function, Tajik, cross-sectional study

## Abstract

**Background:**

Executive function has a significant impact on adolescents’ academic and future achievement and is strongly associated with multiple factors. However, few studies have examined the association between sugar-sweetened beverage (SSB) consumption, cardiorespiratory fitness, and executive function. Moreover, no research studies have been found on Tajik adolescents at high altitudes in China. The present study aimed to understand the associations between SSB consumption, cardiorespiratory fitness, and executive function among Tajik adolescents in high-altitude areas of China. To accumulate information on the physical and mental health development of Tajik adolescents in high-altitude areas of China.

**Methods:**

In this study, a cross-sectional assessment of SSB consumption, cardiorespiratory fitness, and executive function was conducted on 1,121 Tajik adolescents aged 13–15 years at high altitude in China in 2023. One-way analysis of variance (ANOVA), binary logistic regression analysis, and ordered logistic regression analysis with a generalized linear model were used to analyze the associations that existed between SSB consumption and cardiorespiratory fitness and executive function.

**Results:**

The proportions of Tajik adolescents aged 13–15 years with SSB consumption of ≤1 time/week, 2–5 times/week, and ≥ 6 times/week were 14.6, 51.6, and 33.8%, respectively, in high-altitude areas of China. The VO_2max_ of Tajik adolescents was (37.17 ± 5.52) ml.kg.min^−1^. The inhibit control function response, refreshing memory function response, and switching flexibility function response of Chinese Tajik adolescents were (19.71 ± 5.86) ms, (1114.39 ± 356.85) ms, (382.2 ± 213.4) ms. Overall, using the SSB consumption ≤1 times/w and VO_2max_ of the Q4 group as the reference group, ordered logistic regression analysis showed that Tajik adolescents with SSB consumption ≥5 times/w and VO_2max_ of the Q1 group experienced inhibit control function dysfunction (OR = 28.80, 95%CI: 10.23 ~ 81.07), refreshing memory function dysfunction (OR = 6.79, 95%CI: 3.19 ~ 14.43), switching flexibility function dysfunction (OR = 13.10, 95%CI: 5.59 ~ 30.70) were at increased risk (*p* < 0.001).

**Conclusion:**

SSB consumption and cardiorespiratory fitness were associated with executive function in Tajik adolescents at high altitudes in China. Increased frequency of SSB consumption and decreased cardiorespiratory fitness increased the risk of executive function disorders in Tajik adolescents. In the future, SSB consumption and cardiorespiratory fitness should be effectively controlled in Tajik adolescents to improve their executive function and promote the physical and mental health of Tajik adolescents in high-altitude areas.

## 1 Introduction

Executive function, as the core control center of the brain, has an important role and significance in emotional and behavioral control, memory, and cognition (1, 2). Research confirms that adolescents’ levels of executive functioning will be directly related to academic performance, mental health, and future achievement in adulthood, with important roles and significance for healthy development throughout the life cycle (3, 4). It can be seen that executive function plays an important role in the healthy development and future achievement of adolescents, which should be worthy of attention and emphasis. Many studies have confirmed that adolescent executive function is affected by a combination of factors, including family environment, physical fitness level, sleep quality, dietary behavior, brain structure, and living environment (5–7). However, although research on adolescent executive function has been increasing in recent years, previous studies have focused more on the association of factors such as physical exercise, sleep, and the environment with executive function, and more studies have been conducted on special groups of adolescents, such as adolescents with attention-deficit hyperactivity disorder (8, 9). Relatively few studies have been conducted on SSB consumption and cardiorespiratory fitness metrics reflecting the core of physical fitness with executive function.

Reports from the World Health Organization (WHO) show that SSB consumption has shown an increasing trend in recent years in countries around the world and is seriously affecting people’s health (10). A survey shows that SSB consumption among adolescents in various countries is steadily increasing, with serious negative consequences for health (11). China, a developing country, is no exception (12). SSB consumption among Chinese adolescents shows an increasing trend and calls for joint family and school attention to reduce adolescent SSB consumption (13). This shows that the control of SSB consumption and related research needs to be strengthened to remind people to reduce SSB consumption. Studies have shown that increased SSB consumption will lead to chronic cardiovascular disease, type 2 diabetes, obesity, and cancer, resulting in a serious disease burden for all countries and becoming a major common problem (14–18). It has also been shown that increased SSB consumption leads to hormone disruption in the brain, resulting in endocrine disruption and changes in gut flora, which can affect brain health and cognitive development (19, 20). Past studies have addressed the association between SSB consumption and executive function, and better especially for adolescents. However, adolescents are the main group of SSB consumption. A survey of Chinese adolescents showed that increased SSB consumption was associated with decreased levels of executive function (21). It is noteworthy that past studies have focused on adolescent populations in the plains and even fewer related research studies have been conducted on high-altitude areas or ethnic minority groups.

Cardiorespiratory fitness, as an important dimension of physical fitness, has a significant impact on adolescent health. Research has confirmed that the level of cardiorespiratory fitness in adolescents is directly related to academic performance, cardiovascular health, and mental health (22–26). Unfortunately, with changing lifestyles, decreasing MVPA hours, and increasing time spent in static behaviors, cardiorespiratory fitness in adolescents shows a downward trend and has important negative effects on their physical and mental health (27). A study found that cardiorespiratory fitness in adolescents shows a decreasing trend and negatively affects executive function, suggesting that measures should be taken to effectively increase cardiorespiratory fitness levels in adolescents (28). Cardiorespiratory fitness is affected by several factors, in addition to the level of exercise, lifestyle, and muscle strength, there is also a correlation with altitude (29). Some studies have shown that as altitude increases, people’s maximal oxygen uptake level tends to decrease, leading to insufficient oxygen supply to the brain and affecting brain functions (30). High altitude areas have a low oxygen supply due to the scarcity of oxygen, leading to a series of negative problems (31). It has also been shown that people’s executive function levels tend to decrease with increasing altitude and that there is a strong correlation with insufficient oxygen supply to the brain (32).

The Tajiks are the only white people in China (33). This ethnic group lives mainly in the Tashkurgan region of Xinjiang, China. The average altitude of the Tashkurgan region in Xinjiang, China, is over 4,000 meters above sea level, making it a typical high-altitude region (34). It is not clear what the status of SSB consumption, cardiorespiratory fitness, and executive function is in Tajik adolescents living permanently in this region. It is also unclear whether there is an association between SSB consumption and cardiorespiratory fitness and executive function in Tajik adolescents at high altitudes. For this reason, the present study was conducted to assess SSB consumption, cardiorespiratory fitness, and executive function in a cross-sectional sample of 1,121 Tajik adolescents aged 13–15 years in this region. The aim was to understand the associations that exist between SSB consumption, cardiorespiratory fitness, and executive function in Tajik adolescents in high-altitude areas of China. It will help the physical and mental health development of Tajik adolescents in high-altitude areas of China.

## 2 Methods

### 2.1 Participants

In this study, 1,121 Tajik adolescents aged 13–15 years were assessed cross-sectionally for SSB consumption, cardiorespiratory fitness, and executive function from April to July 2023 in Tashkurgan County, a high-altitude area of China. The 13-15-year-old Chinese Tajik adolescents in this study were in the middle school stage, a stage where students study in a boarding school and are away from their parents’ attention. In addition, 13-15-year-old adolescents are in the critical period of cardiorespiratory fitness and executive function development, and research on this group is of value and significance. Participant extraction for this study was divided into the following three main stages: First, Tashkurgan in Xinjiang, China, where Tajiks are more concentrated, was used as the survey area for the participants of this study. Tashkurgan County has an average elevation of over 4,000 meters above sea level, which is a typical high-altitude area. Second, two middle schools were randomly selected among all middle schools in Tashkurgan County as the survey schools for this study. Third, in the selected schools, 4 teaching classes were randomly selected at each grade level, and Tajik middle school students in the classes who met the conditions of this study served as participants.

Participants in this study were included under the following conditions: adolescents of Tajik ethnicity who were born in the Tashkurgan region of China and have been growing up in the region; the age requirement was between 13 and 15 years old; the participants’ fathers and mothers were of Tajik ethnicity; and the participants themselves and their guardians gave informed consent and voluntarily participated in the evaluation of this survey. This study was conducted by the Declaration of Helsinki, and approved by the Human Ethics Committee of Shihezi University (202335472).

**Figure 1:**
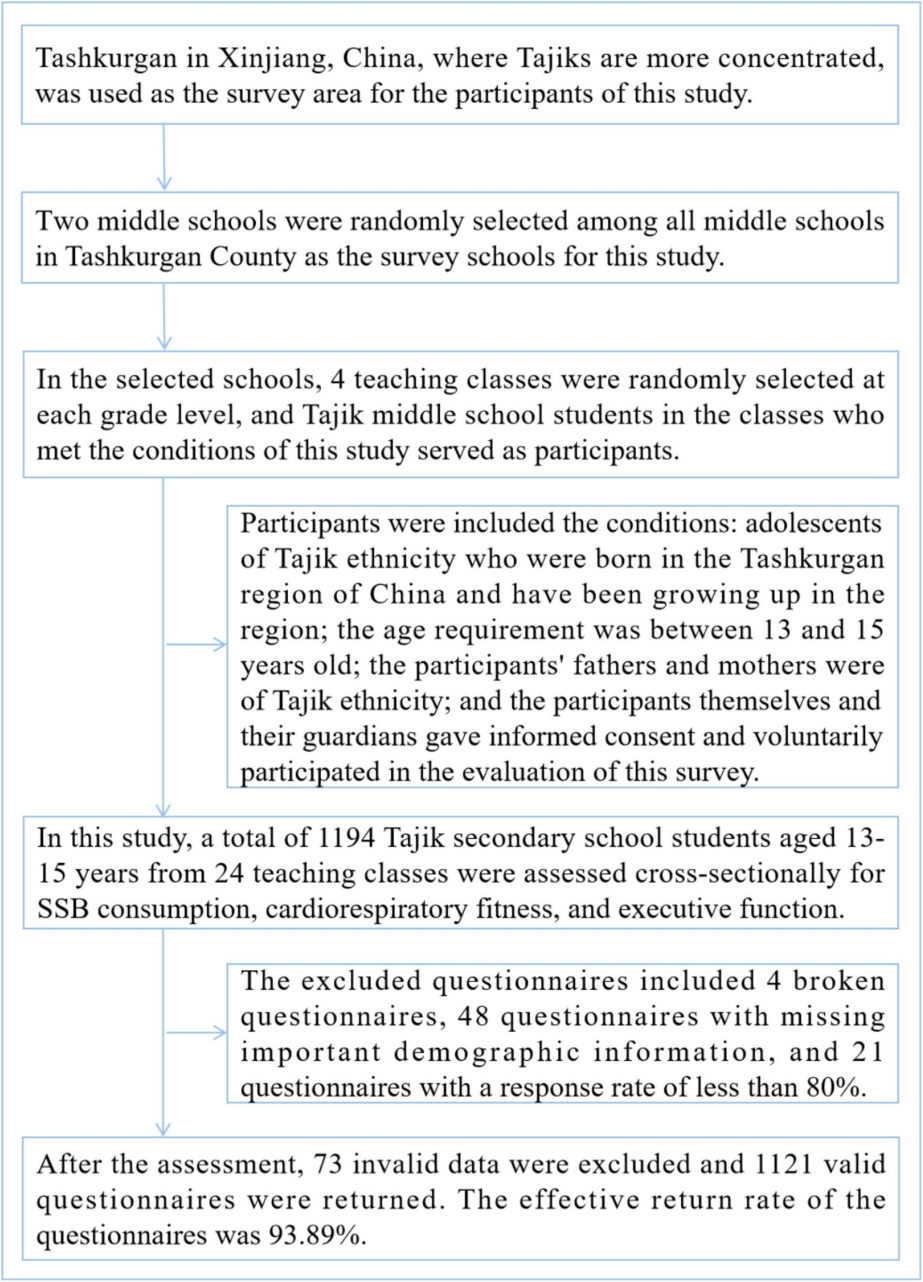
Sampling flow of adolescent participants of Tajik ethnicity in high-altitude areas of Xinjiang, China.

In this study, a total of 1,194 Tajik secondary school students aged 13–15 years from 24 teaching classes were assessed cross-sectionally for SSB consumption, cardiorespiratory fitness, and executive function. After the assessment, 73 invalid data were excluded and 1,121 valid questionnaires were returned. The effective return rate of the questionnaires was 93.89%. The excluded questionnaires included 4 broken questionnaires, 48 questionnaires with missing important demographic information, and 21 questionnaires with a response rate of less than 80%. Figure 1 shows the sampling process of participants in this study.

### 2.2 SSB consumption assessment

In this study, SSB consumption was assessed using the internationally used beverage intake questionnaire (BEVQ-15) scale (35). The scale consists of 15 entries and was used to assess participants’ SSB consumption over the past 1 month. Specifically, it includes the frequency of participants’ SSB consumption in the past 1 month, the number of milliliters per occasion, and the type of SSB consumed. In past studies, the BEVQ-15 scale has been commonly used among Chinese adolescents with good reliability and validity (36, 37). The types of SSB consumption mainly include functional drinks, carbonated drinks, coffee, beer, wine, nut-based drinks, nut-based milk, fruit juices with added sugar, unsweetened beverages, milk with added sugar, milk tea, and other beverages with added sugar that are not involved. Participants were asked to fill in the appropriate type of SSB and frequency and milliliters according to their reality. The frequency of the BEVQ-15 scale was categorized as “3 or more times/day,” “2 times/day,” “1 time/day,” “4–6 times/week,” “2–3 times/week,” “1 time/week,” and “never or less than 1 time/week.” The number of milliliters consumed by SSB was categorized as “20 ounces and above,” “20 ounces (2.5 cups),” “16 ounces (2 cups),” “12 ounces (1.5 cups),” ‘8 ounces (1 cup)’, and ‘less than 6 ounces’. The final calculations were based on the actual information filled out by the participants, using the formula established by the BEVQ-15 (38). In this study, to facilitate the investigation of Tajik adolescents, based on the local situation, the study was based on the consumption of “8 ounces (1 cup)” per time as the calculation standard. The frequency of SSB consumption in the past 30 days was categorized as “≤1 times/week,” “2–5 times/week,” and “≥6 times/week”.

### 2.3 Cardiorespiratory fitness assessment

The assessment of cardiorespiratory fitness in this study was based on the test items required by the China National Survey on Students’ Constitution and Health (CNSSCH) (39). Cardiorespiratory fitness was assessed using a 1,000-meter run for males and an 800-meter run for females. The participant’s performance was evaluated in seconds, and the specific performance was accurate to 1 s. Based on the participants’ final assessment scores, the VO_2max_ of the participants was calculated using an extrapolation formula developed for Chinese adolescents, which was as follows: VO_2max_ (L/min) = 1.640–0.004 × gender (male 1, female 2) × time (s) + 0.037 × body weight (kg) (40). Weight was also assessed by following the instruments and assessment methods required by the CNSSCH. Before the weight assessment, participants were asked to empty their bowels and urine while wearing light clothing for the assessment. Participants were asked to fully prepare for the 1,000-meter run (boys) and 800-meter run (girls) before the assessment to avoid the occurrence of sports injuries.

### 2.4 Executive function assessment

The assessment of executive function in this study included inhibitory control function, refresh memory function and conversion flexibility function. (1) The inhibitory control function mainly assesses the participant’s ability to control their emotions, attention, thoughts, and behavioral habits to control or suppress strong internal tendencies or external temptations of various kinds to achieve the ability to carry out all kinds of tasks correctly, reasonably, and rationally. (2) The refreshing memory function, also known as the working memory capacity, usually refers to the ability to deeply memorize external information in the brain in a relatively short period and to carry out rapid information processing and processing. It mainly assesses the ability of the participant’s brain to memorize relevant information in a very short period and perform rapid extraction and processing of such information. (3) Switching Flexibility Function refers to the ability to constantly switch or switch between multiple tasks, also known as cognitive flexibility. Switching flexibility function can better reflect the participants’ ability to quickly adjust their cognition and behavior to adapt to the changing environment faster.

Participants’ inhibitory control function, refreshing memory function, and switching flexibility function in this study were assessed using the Flanker experimental paradigm, the 2-back experimental paradigm, and the More-odd Shifting experimental paradigm, respectively. All three paradigms are internationally used experimental paradigms, which have been adopted in several studies and have good reliability and validity for assessing adolescents’ executive function. The paradigms were pre-installed on a computer, and participants were centrally assessed in a school computer classroom. The assessment was conducted independently in a quiet and brightly lit environment. The assessment program was based on the E-prime 1.1 computer software system. The flanker experimental paradigm, 2-back experimental paradigm, and more-odd Shifting experimental paradigm were assessed in a manner consistent with previously published literature (28).

### 2.5 Covariates

The covariates in this study included father’s education, mother’s education, smoking, adequacy of sleep, body mass index (BMI), grip strength, Moderate to vigorous physical activity (MVPA). Father’s education and mother’s education are categorized as “Elementary school and below,” “Middle or High School,” “College and above.” “Smoking” was categorized as “No” and “Yes.” Adequacy of sleep was categorized as “Adequacy (≥8 h/d)” and “Insufficient (<8 h/d),” and BMI was calculated based on participants’ height and weight. Height and weight were assessed according to the test methods and instruments required by the CNSSCH (39). Height was measured to the nearest 0.1 cm and weight to the nearest 0.1 kg. BMI = weight (kg)/height (m)^2^. Grip strength was assessed using a grip strength meter. Participants were asked to perform the test twice with a strong hand, and the better score was taken as the participant’s test score. MVPA was assessed based on the questionnaire in CNSSCH (39). Participants were assessed on the average number of hours of MVPA participation per day over the past 7 days. The participant group filled in the average length of MVPA per day on weekdays from Monday to Friday, as well as the average length of MVPA per day on Saturdays and weekdays. From this, the average length of time that participants participated in MVPA per day on average over the past 7 days was calculated.

### 2.6 Statistical analysis

The basic status of the continuous type of the participants in this study was expressed using mean and standard deviation. Subtypes of conditions were expressed as percentages. Comparisons between groups of basic conditions were made using one-way ANOVA and chi-square tests. Comparisons between groups with different SSB consumption and different VO_2 max_ at the time of executive function response were made by one-way ANOVA. Participants’ VO_2max_ was divided into four groups, Q1, Q2, Q3, and Q4, based on the stratification of different ages and genders followed by the division of quartiles. In this study, participants’ executive function dysfunction was stratified by age and sex and then classified according to the mean value. When the participants’ executive function response time was higher than the mean, it was defined as the presence of the corresponding “function dysfunction.” The associations of SSB consumption, cardiorespiratory fitness, and executive function were analyzed using binary logistic regression analysis and ordered logistic regression analysis with generalized linear models. Model 1 was the crude model for binary logistic regression analysis, and Model 2 was adjusted for age, father’s education, mother’s education, smoking, adequacy of sleep, BMI, grip strength, and MVPA based on Model 1. When conducting the ordered logistic regression analysis of the generalized linear model, the model analyzed age, father’s education, mother’s education, smoking, adequacy of sleep, BMI, grip strength, and MVPA duration as covariates.

## 3 Results

In this study, SSB consumption and cardiorespiratory fitness and executive function were assessed in 1121 Tajik adolescents aged 13–15 years in the high-altitude region of Xinjiang, China. The mean age of the participants was (13.95 ± 0.81) years. The proportions of Tajik adolescents with SSB consumption of ≤1 times/week, 2–5 times/week, and ≥ 6 times/week in this study were 14.6, 51.6, and 33.8%, respectively. The VO_2max_ of Tajik adolescents at high altitude was (37.17 ± 5.52) ml.kg.min-1; among them, the VO_2max_ of boys (39.06 ± 6.34) was higher than that of girls (35.31 ± 3.73), with a statistically significant difference (t-value of 12.095, *p* < 0.001). The inhibit control function response, refreshing memory function response, and switching flexibility function response of Tajik adolescents at high altitude in Xinjiang, China, were (19.71 ± 5.86) ms, (1114.39 ± 356.85) ms, and (382.2 ± 213.4) ms, respectively. The basic conditions of Tajik adolescents in high-altitude areas of Xinjiang, China, are shown in Table 1.

**Table 1:** Basic situation of Tajik adolescents in high-altitude areas of Xinjiang, China.

Items	Boys	Girls	*χ*^2^-value/*t*-value	*p*-value	Total
Number	555	566			1,121
Age (years)	13.97 ± 0.82	13.93 ± 0.81	0.714	0.475	13.95 ± 0.81
Father’s education			5.605	0.061	
Elementary school and below	290 (59.1)	322 (66.4)			612 (62.7)
Middle or High School	136 (27.7)	110(22.7)			246 (25.2)
College and above	65 (13.2)	53 (10.9)			118 (12.1)
Mother’s education			6.938	0.031	
Elementary school and below	327 (68.3)	374 (75.4)			701 (71.9)
Middle or High School	84 (17.5)	74 (14.9)			158 (16.2)
College and above	68 (14.2)	48 (9.7)			116 (11.9)
Smoking			0.062	0.804	
No	200 (36.0)	208 (36.7)			408 (36.4)
Yes	355 (64.0)	358 (63.3)			713 (63.6)
Adequacy of sleep			51.341	<0.001	
Adequacy (≥8 h/d)	303 (55.3)	191 (33.9)			494 (44.5)
Insufficient (<8 h/d)	245 (44.7)	372 (66.1)			617 (55.5)
SSB consumption			19.746	<0.001	
≤1 times/week	93 (16.8)	71 (12.5)			164 (14.6)
2–5 times/week	249 (44.9)	329(58.1)			578 (51.6)
≥6 times/week	213 (38.4)	166 (29.3)			379 (33.8)
VO_2max_ Quartile			273.811	<0.001	
Q1	133 (24.0)	161 (28.4)			294 (26.2)
Q2	53 (9.5)	244 (43.1)			297 (26.5)
Q3	134 (24.1)	126 (22.3)			260 (23.2)
Q4	235 (42.3)	35 (6.2)			270 (24.1)
Inhibit control function dysfunction			0.478	0.489	
No	287 (51.7)	281 (49.6)			568 (50.7)
Yes	268 (48.3)	285 (50.4)			553 (49.3)
Refreshing memory function dysfunction			1.329	0.249	
No	279 (50.3)	304 (53.7)			583 (52.0)
Yes	276 (49.7)	262 (46.3)			538 (48.0)
Switching flexibility function dysfunction			0.002	0.963	
No	314 (56.6)	321 (56.7)			635 (56.6)
Yes	241 (43.4)	245 (43.3)			486 (43.4)
BMI (kg/m^2^)	19.95 ± 2.37	21.03 ± 2.26	7.837	<0.001	20.49 ± 2.38
Grip strength (kg)	37.67 ± 10.20	27.51 ± 5.74	20.590	<0.001	32.54 ± 9.69
MVPA (min/day)	54.87 ± 44.66	29.84 ± 29.81	11.054	<0.001	42.23 ± 39.89
VO_2max_ (ml.kg.min^−1^)	39.06 ± 6.34	35.31 ± 3.73	12.095	<0.001	37.17 ± 5.52
Inhibit control function response (ms)	19.84 ± 5.80	19.59 ± 5.92	0.711	0.477	19.71 ± 5.86
Refreshing memory function response (ms)	1137.81 ± 346.62	1091.42 ± 365.47	2.180	0.029	1114.39 ± 356.85
Switching flexibility function response (ms)	380.44 ± 222.26	383.92 ± 204.51	−0.272	0.786	382.20 ± 213.40

BMI, Body mass index; MVPA, Moderate to vigorous physical activity; SSB, sugar-sweetened beverage; VO_2max_, maximal oxygen consumption. “()” in %.

Table 2 shows the one-way ANOVA for SSB consumption and cardiorespiratory fitness and executive function in Tajik adolescents at high altitudes in Xinjiang, China. The results showed that in terms of different SSB consumption, boys, girls, and overall, Tajik adolescents’ inhibit control function, refreshing memory function, and switching flexibility function response times were compared with each other, and the differences were statistically significant (*p* < 0.001). In terms of different VO_2max_, the differences were statistically significant (*p* < 0.05) when comparing the response times of inhibit control function, refreshing memory function, and switching flexibility function among Tajik adolescents in boys, girls, and overall. Overall, the adolescents with higher SSB consumption had a longer response time for executive function, indicating a poorer level of executive function. In addition, adolescents with higher VO_2max_ (Q4) had a shorter executive function reaction time, indicating a better level of executive function.

**Table 2:** One-way ANOVA for SSB consumption and cardiorespiratory fitness with executive function in Tajik adolescents at high altitude in Xinjiang, China.

Sex/Category	Group	Number	Inhibit control function			Refreshing memory function			Switching flexibility function		
			M (SD)	*F*-value	*p*-value	M (SD)	*F*-value	*p*-value	M (SD)	*F*-value	*p*-value
Boys											
SSB	≤1 times/week	93	15.19 ± 3.93	55.156	<0.001	1007.82 ± 382.59	16.902	<0.001	265.80 ± 188.20	29.763	<0.001
	2–5 times/week	249	19.63 ± 5.69			1103.84 ± 368.44			356.47 ± 238.62		
	≥6 times/week	213	22.10 ± 5.37			1234.28 ± 271.16			458.52 ± 185.87		
VO_2max_	Q1	133	21.49 ± 5.78	8.969	<0.001	1289.85 ± 282.37	32.819	<0.001	509.80 ± 273.71	44.194	<0.001
	Q2	53	21.44 ± 5.73			1321.61 ± 286.50			530.81 ± 230.47		
	Q3	134	19.75 ± 6.25			1171.80 ± 325.90			340.36 ± 187.38		
	Q4	235	18.59 ± 5.26			990.92 ± 343.12			296.18 ± 142.31		
Girls											
SSB	≤1 times/week	71	15.42 ± 3.20	45.929	<0.001	913.41 ± 330.05	19.862	<0.001	291.15 ± 169.36	24.479	<0.001
	2–5 times/week	329	19.00 ± 6.41			1067.48 ± 392.30			362.36 ± 215.09		
	≥6 times/week	166	22.53 ± 4.14			1215.00 ± 273.98			466.31 ± 166.23		
VO_2max_	Q1	161	20.68 ± 6.33	2.672	0.047	1169.73 ± 361.1	3.991	0.008	454.53 ± 258.26	10.166	<0.001
	Q2	244	19.20 ± 5.86			1077.96 ± 382.51			361.01 ± 174.75		
	Q3	126	19.21 ± 5.73			1035.66 ± 343.86			359.61 ± 171.89		
	Q4	35	18.62 ± 4.48			1025.75 ± 281.39			306.24 ± 136.11		
Total											
SSB	≤1 times/week	164	15.29 ± 3.62	99.781	<0.001	966.95 ± 362.76	36.922	<0.001	276.78 ± 180.19	54.378	<0.001
	2–5 times/week	578	19.27 ± 6.11			1083.14 ± 382.30			359.82 ± 225.34		
	≥6 times/week	379	22.29 ± 4.87			1225.83 ± 272.20			461.93 ± 177.35		
VO_2max_	Q1	294	21.05 ± 6.09	8.698	<0.001	1224.07 ± 332.74	20.349	<0.001	479.54 ± 266.33	40.673	<0.001
	Q2	297	19.60 ± 5.89			1121.44 ± 378.51			391.31 ± 196.58		
	Q3	260	19.49 ± 6.00			1105.83 ± 340.96			349.69 ± 179.95		
	Q4	270	18.59 ± 5.16			995.43 ± 335.50			297.48 ± 141.31		

M, mean; SD, standard deviation; SSB, sugar-sweetened beverage; VO_2max_, maximal oxygen consumption.

Table 3 shows the binary logistic regression analysis of SSB consumption and cardiorespiratory fitness with executive function in Tajik adolescents in high-altitude areas of Xinjiang, China. Binary logistic regression analysis was performed with the presence of executive function disorder as the dependent variable and SSB consumption and VO_2max_ as the independent variables. Model 1 was the crude model, and Model 2 was adjusted for age, father’s education, mother’s education, smoking, adequacy of sleep, BMI, grip strength, and MVPA based on Model 1. Logistic regression analyses showed that after adjusting for relevant covariates, overall, adolescents with SSB consumption ≤1 time/week were analyzed as the reference group. Adolescents with SSB consumption of 2–5 times/week developed inhibit control function dysfunction (OR = 8.60, 95%CI: 4.58 ~ 16.16), refreshing memory function dysfunction (OR = 1.51, 95%CI: 0.99 ~ 2.31), and increased risk of switching flexibility function dysfunction (OR = 2.04, 95%CI: 1.29 ~ 3.23). Adolescents with SSB consumption ≥6 times/week developed inhibit control function dysfunction (OR = 23.42, 95%CI: 12.16 ~ 45.11), refreshing memory function dysfunction (OR = 2.57, 95%CI: 1.64 ~ 4.02), and switching flexibility function dysfunction (OR = 4.28, 95%CI: 2.65 ~ 6.91) were also at increased risk (*p* < 0.001). Overall, using adolescents with a VO_2max_ of Q4 as the reference group, adolescents with a VO_2max_ of Q1 had an increased risk of refreshing memory function dysfunction (OR = 3.65, 95%CI: 2.33 ~ 5.71), switching flexibility function dysfunction (OR = 4.36, 95%CI: 2.76 ~ 6.88) were also at increased risk (*p* < 0.001).

**Table 3:** Binary logistic regression analysis of SSB consumption and cardiorespiratory fitness with executive function disorder in Tajik adolescents at high altitude in Xinjiang, China.

Sex/Variable	Group	Inhibit control function dysfunction				Refreshing memory function dysfunction				Switching flexibility function dysfunction			
		Model 1		Model 2		Model 1		Model 2		Model 1		Model 2	
		OR (95% CI)	*p-*value	OR (95% CI)	*p-*value	OR (95% CI)	*p-*value	OR (95% CI)	*p-*value	OR (95% CI)	*p-*value	OR (95% CI)	*p-*value
Boys													
SSB	≤1 times/week	1.00		1.00		1.00		1.00		1.00		1.00	
	2–5 times/week	16.44 (5.80 ~ 46.55)	<0.001	16.96 (5.86 ~ 49.11)	<0.001	1.49 (0.87 ~ 2.55)	0.151	1.30 (0.72 ~ 2.35)	0.382	2.23 (1.23 ~ 4.04)	0.008	2.11 (1.14 ~ 3.92)	0.018
	≥6 times/week	36.33 (12.61 ~ 104.70)	<0.001	34.12 (11.55 ~ 100.77)	<0.001	2.88 (1.64 ~ 5.07)	<0.001	2.46 (1.32 ~ 4.57)	0.005	4.28 (2.32 ~ 7.90)	<0.001	3.93 (2.06 ~ 7.48)	<0.001
VO_2max_	Q4	1.00		1.00		1.00		1.00		1.00		1.00	
	Q3	1.44 (0.90 ~ 2.31)	0.127	1.10 (0.65 ~ 1.84)	0.729	3.16 (1.95 ~ 5.14)	<0.001	2.61 (1.54 ~ 4.42)	<0.001	1.75 (1.07 ~ 2.88)	0.027	1.80 (1.05 ~ 3.07)	0.031
	Q2	2.19 (1.12 ~ 4.29)	0.022	1.45 (0.7 ~ 2.99)	0.314	6.46 (3.10 ~ 13.47)	<0.001	4.80 (2.21 ~ 10.44)	<0.001	7.15 (3.42 ~ 14.96)	<0.001	7.15 (3.29 ~ 15.57)	<0.001
	Q1	2.25 (1.39 ~ 3.62)	0.001	1.41 (0.82 ~ 2.45)	0.215	6.02 (3.59 ~ 10.08)	<0.001	4.07 (2.29 ~ 7.22)	<0.001	6.11 (3.65 ~ 10.22)	<0.001	5.86 (3.28 ~ 10.48)	<0.001
Girls													
SSB	≤1 times/week	1.00		1.00		1.00		1.00		1.00		1.00	
	2–5 times/week	5.33 (2.43 ~ 11.68)	<0.001	5.17 (2.32 ~ 11.51)	<0.001	1.79 (0.97 ~ 3.32)	0.063	1.80 (0.95 ~ 3.40)	0.071	2.32 (1.17 ~ 4.60)	0.015	2.18 (1.08 ~ 4.41)	0.030
	≥6 times/week	20.51 (8.81 ~ 47.73)	<0.001	20.46 (8.51 ~ 49.17)	<0.001	2.59 (1.35 ~ 5.01)	0.004	2.31 (1.16 ~ 4.60)	0.018	5.22 (2.54 ~ 10.73)	<0.001	4.70 (2.2 ~ 10.02)	<0.001
VO_2max_	Q4	1.00		1.00		1.00		1.00		1.00		1.00	
	Q3	1.24 (0.53 ~ 2.91)	0.620	1.13 (0.46 ~ 2.76)	0.786	1.30 (0.52 ~ 3.27)	0.579	1.25 (0.48 ~ 3.27)	0.643	2.09 (0.77 ~ 5.65)	0.147	1.91 (0.68 ~ 5.35)	0.217
	Q2	0.88 (0.40 ~ 1.97)	0.761	0.83 (0.36 ~ 1.94)	0.672	1.66 (0.69 ~ 3.96)	0.257	1.62 (0.65 ~ 4.02)	0.297	2.02 (0.78 ~ 5.24)	0.147	1.76 (0.66 ~ 4.72)	0.258
	Q1	1.56 (0.68 ~ 3.57)	0.297	1.43 (0.59 ~ 3.47)	0.434	3.13 (1.28 ~ 7.65)	0.013	3.21 (1.24 ~ 8.29)	0.016	4.47 (1.69 ~ 11.78)	0.003	3.39 (1.23 ~ 9.38)	0.019
Total													
SSB	≤1 times/week	1.00		1.00		1.00		1.00		1.00		1.00	
	2–5 times/week	8.95 (4.82 ~ 16.62)	<0.001	8.60 (4.58 ~ 16.16)	<0.001	1.59 (1.06 ~ 2.38)	0.024	1.51 (0.99 ~ 2.31)	0.055	2.23 (1.43 ~ 3.49)	<0.001	2.04 (1.29 ~ 3.23)	0.002
	≥6 times/week	25.30 (13.29 ~ 48.15)	<0.001	23.42 (12.16 ~ 45.11)	<0.001	2.71 (1.77 ~ 4.16)	<0.001	2.57 (1.64 ~ 4.02)	<0.001	4.66 (2.92 ~ 7.43)	<0.001	4.28 (2.65 ~ 6.91)	<0.001
VO_2max_	Q4	1.00		1.00		1.00		1.00		1.00		1.00	
	Q3	1.54 (1.05 ~ 2.25)	0.028	1.10 (0.73 ~ 1.67)	0.647	2.13 (1.43 ~ 3.17)	<0.001	1.77 (1.15 ~ 2.73)	0.009	1.75 (1.16 ~ 2.64)	0.007	1.74 (1.11 ~ 2.70)	0.015
	Q2	1.35 (0.94 ~ 1.95)	0.106	0.86 (0.56 ~ 1.31)	0.474	2.16 (1.47 ~ 3.17)	<0.001	1.75 (1.13 ~ 2.70)	0.012	2.13 (1.44 ~ 3.16)	<0.001	2.09 (1.33 ~ 3.26)	0.001
	Q1	2.16 (1.49 ~ 3.13)	<0.001	1.43 (0.93 ~ 2.20)	0.104	4.24 (2.87 ~ 6.27)	<0.001	3.65 (2.33 ~ 5.71)	<0.001	4.61 (3.10 ~ 6.85)	<0.001	4.36 (2.76 ~ 6.88)	<0.001

OR, odds ratio; 95%CI, confidence interval; SSB, sugar-sweetened beverage; VO2max, maximal oxygen consumption. Model 1 is the crude model and Model 2 adjusts for age, father’s education, mother’s education, smoking, adequacy of sleep, BMI, grip strength, and MVPA based on Model 1.

Table 4 shows the ordered logistic regression analysis of SSB consumption and cardiorespiratory fitness with executive function in Tajik adolescents in high-altitude areas of Xinjiang, China. Ordered logistic regression analyses were performed with the presence of executive function impairment as the dependent variable and SSB consumption and VO_2max_ as the independent variables. The model was adjusted for age, father’s education, mother’s education, smoking, adequacy of sleep, BMI, grip strength, and MVPA. The results showed that overall, using the group with SSB consumption ≤1 times/w and VO_2max_ of Q4 as the reference group, ordered logistic regression analysis showed that adolescents in the group with SSB consumption ≥5 times/w and VO_2max_ of Q1 developed inhibit control function dysfunction (OR = 28.80, 95%CI: 10.23 ~ 81.07), refreshing memory function dysfunction (OR = 6.79, 95%CI: 3.19 ~ 14.43), switching flexibility function dysfunction (OR = 13.10, 95%CI: 5.59 ~ 30.70) were at increased risk (*p* < 0.001).

**Table 4:** Ordered logistic regression analysis of SSB consumption and cardiorespiratory fitness with executive function in Tajik adolescents at high altitude in Xinjiang, China.

Sex	Classification of interaction		Inhibit control function dysfunction		Refreshing memory function dysfunction		Switching flexibility function dysfunction	
	SSB	VO_2max_	OR (95% CI)	*p*-value	OR (95% CI)	*p*-value	OR (95% CI)	*p*-value
Boys	≤1 times/w	Q4	1.00		1.00		1.00	
		Q3	–		1.74 (0.52 ~ 5.87)	0.370	0.88 (0.16 ~ 4.77)	0.881
		Q2	–		3.92 (0.59 ~ 26.23)	0.159	22.86 (2.22 ~ 235.82)	0.009
		Q1	1.83 (0.17 ~ 19.91)	0.620	3.27 (0.76 ~ 14.1)	0.112	7.14 (1.53 ~ 33.34)	0.012
	2–4 times/w	Q4	14.01 (4.05 ~ 48.53)	<0.001	0.72 (0.32 ~ 1.63)	0.431	1.79 (0.70 ~ 4.59)	0.225
		Q3	7.93 (2.18 ~ 28.80)	<0.001	3.35 (1.47 ~ 7.65)	0.004	2.86 (1.08 ~ 7.56)	0.035
		Q2	19.07 (4.56 ~ 79.74)	<0.001	4.90 (1.68 ~ 14.3)	0.004	10.71 (3.31 ~ 34.7)	<0.001
		Q1	18.49 (5.08 ~ 67.26)	<0.001	4.54 (1.94 ~ 10.66)	0.001	11.76 (4.37 ~ 31.67)	<0.001
	≥5 times/w	Q4	14.19 (3.98 ~ 50.68)	<0.001	1.70 (0.75 ~ 3.85)	0.206	3.71 (1.43 ~ 9.62)	0.007
		Q3	80.67 (18.78 ~ 346.5)	<0.001	4.18 (1.69 ~ 10.38)	0.002	7.39 (2.66 ~ 20.56)	<0.001
		Q2	58.67 (10.47 ~ 328.58)	<0.001	17.00 (3.36 ~ 85.91)	0.001	22.86 (5.11 ~ 102.28)	<0.001
		Q1	35.62 (9.47 ~ 133.98)	<0.001	11.33 (4.31 ~ 29.79)	<0.001	15.38 (5.52 ~ 42.87)	<0.001
Girls	≤1 times/w	Q4	1.00		1.00		1.00	
		Q3	1.33 (0.16 ~ 11.07)	0.790	0.50 (0.05 ~ 4.98)	0.554	0.22 (0.02 ~ 3.22)	0.270
		Q2	0.22 (0.03 ~ 1.58)	0.131	0.93 (0.15 ~ 5.73)	0.936	0.56 (0.09 ~ 3.58)	0.542
		Q1	–		4.00 (0.21 ~ 75.66)	0.355	4.00 (0.21 ~ 75.66)	0.355
	2–4 times/w	Q4	1.75 (0.24 ~ 12.64)	0.579	1.33 (0.18 ~ 9.72)	0.777	0.50 (0.06 ~ 4.15)	0.521
		Q3	1.27 (0.21 ~ 7.57)	0.791	–		0.84 (0.14 ~ 5.07)	0.851
		Q2	1.63 (0.29 ~ 9.29)	0.580	2.25 (0.39 ~ 12.95)	0.364	1.11 (0.20 ~ 6.36)	0.903
		Q1	2.05 (0.36 ~ 11.78)	0.422	2.25 (0.39 ~ 12.95)	0.364	1.78 (0.31 ~ 10.23)	0.519
	≥5 times/w	Q4	4.00 (0.36 ~ 44.11)	0.258	–		0.40 (0.03 ~ 6.18)	0.512
		Q3	8.00 (1.21 ~ 52.88)	0.031	1.50 (0.24 ~ 9.30)	0.663	2.67 (0.43 ~ 16.53)	0.292
		Q2	5.43 (0.89 ~ 32.99)	0.066	2.00 (0.34 ~ 11.89)	0.446	2.00 (0.34 ~ 11.89)	0.446
		Q1	7.78 (1.22 ~ 49.40)	0.030	3.50 (0.58 ~ 21.28)	0.174	5.33 (0.86 ~ 33.00)	0.072
Total	≤1 times/w	Q4	1.00		1.00		1.00	
		Q3	1.83 (0.45 ~ 7.50)	0.402	1.19 (0.43 ~ 3.34)	0.738	0.67 (0.16 ~ 2.71)	0.571
		Q2	0.91 (0.23 ~ 3.63)	0.899	1.35 (0.58 ~ 3.17)	0.489	1.93 (0.74 ~ 5.04)	0.182
		Q1	0.87 (0.09 ~ 8.24)	0.905	3.55 (0.97 ~ 12.94)	0.055	6.84 (1.77 ~ 26.49)	0.005
	2–4 times/w	Q4	9.06 (3.34 ~ 24.59)	<0.001	0.81 (0.38 ~ 1.72)	0.585	1.49 (0.63 ~ 3.48)	0.362
		Q3	5.62 (2.07 ~ 15.26)	0.001	2.08 (1.03 ~ 4.20)	0.042	2.25 (0.99 ~ 5.12)	0.053
		Q2	8.50 (3.18 ~ 22.71)	<0.001	2.05 (1.03 ~ 4.08)	0.042	3.38 (1.53 ~ 7.51)	0.003
		Q1	10.63 (3.99 ~ 28.34)	<0.001	3.35 (1.69 ~ 6.66)	0.001	5.91 (2.68 ~ 13.06)	<0.001
	≥5 times/w	Q4	9.89 (3.50 ~ 27.93)	<0.001	1.61 (0.74 ~ 3.48)	0.230	2.91 (1.22 ~ 6.96)	0.016
		Q3	10.05 (4.02 ~ 23.13)	<0.001	2.82 (1.33 ~ 5.99)	0.007	6.42 (2.74 ~ 15.04)	<0.001
		Q2	28.24 (9.66 ~ 82.56)	<0.001	3.53 (1.63 ~ 7.62)	0.001	6.41 (2.70 ~ 15.21)	<0.001
		Q1	28.80 (10.23 ~ 81.07)	<0.001	6.79 (3.19 ~ 14.43)	<0.001	13.10 (5.59 ~ 30.70)	<0.001

OR, odds ratio; 95%CI, confidence interval; SSB, sugar-sweetened beverage; VO_2max_, maximal oxygen consumption. Age, father’s education, mother’s education, smoking, adequacy of sleep, BMI, grip strength, and MVPA hours were analyzed as covariates in the ordered logistic regression.

## 4 Discussion

To the best of our knowledge, this study is the first to analyze the associations of SSB consumption and cardiorespiratory fitness with executive function in Tajik adolescents at high altitudes in Xinjiang, China. This study found that SSB consumption and cardiorespiratory fitness were associated with executive function in Tajik adolescents. Increased frequency of SSB consumption and decreased cardiorespiratory fitness in adolescents increase the risk for the development of executive function disorders in Tajik adolescents. This study also found that the proportion of Tajik adolescents with SSB consumption of ≤1 time/week, 2–5 times/week, and ≥ 6 times/week in the high-altitude region of Xinjiang, China, was 14.6, 51.6, and 33.8%, respectively. Because the criteria for defining SSB consumption are not consistent across studies, accurate cross-sectional comparative analyses are not possible (41). However, the results of this study showed that the proportion of Tajik adolescents with SSB consumption ≥6 times/week in the high-altitude region of Xinjiang, China, amounted to more than 30%, which is sufficient to indicate that a certain proportion of Tajik adolescents with higher SSB consumption should be given sufficient attention and concern. The present study also found that the VO_2max_ of Tajik adolescents at high altitudes was (37.17 ± 5.52) ml.kg.min^−1^, a result that was lower compared to the findings of Zhang et al. on Chinese adolescents of the same age group (38.3–43.6 mL.kg.min-1) (42). The reasons for this result are multiple. First, the Tajik adolescents investigated in this study were adolescents at high altitude, and as the altitude increases, there is less oxygen in the air, so it is difficult to safeguard the body’s need for oxygen during cardiorespiratory fitness assessment, which leads to lower levels of cardiorespiratory fitness. There may also be a correlation with genetic differences due to differences in ethnic composition. Past studies have found that cardiorespiratory fitness is influenced by genetic factors in addition to acquired exercise (43). Whether there are other reasons or not will require more relevant investigations and studies in the future.

In recent years, the increase in SSB consumption among adolescents has become an important issue shared by all countries (44). In this study, we also found that SSB consumption was associated with executive function in Tajik adolescents in the high-altitude region of Xinjiang, China and that increased SSB consumption led to a higher risk for the development of executive function disorders. Studies show that increased SSB consumption is also an important risk factor for obesity in adolescents (45). It has also been shown that obese individuals have relatively lower levels of executive function compared to normal-weight adolescents (46). We suggest that increased SSB consumption in Tajik adolescents leads to a higher risk of obesity development, which in turn causes a decrease in executive function levels. On the other hand, the increased consumption of the SSB leads to certain disturbances in the intestinal flora of adolescents, which affects the secretion of hormones in the body, causing changes in the “gut-brain axis,” and consequently changes in the executive function. Studies have shown that changes in intestinal flora are important factors that lead to changes in the body’s hormone secretion, which affects the development of the executive function and causes a decline in the executive function (47). Our findings also show that there is an association between reduced levels of cardiorespiratory fitness and increased risk of executive function disorders in Tajik adolescents. It has been found that reduced levels of cardiorespiratory fitness lead to alterations in the hemodynamic characteristics of the participants’ brains, which in turn cause a reduction in executive function (48). It has also been shown that reduced cardiorespiratory fitness leads to lower levels of neuronal regeneration in the brain, which also hurts the development of executive function (49).

Our study also found that Tajik adolescents with higher SSB consumption and lower cardiorespiratory fitness were at higher risk for executive function disorders. The reason for this is that adolescents with higher SSB consumption tend to have higher body weights or are overweight and obese, and have less time for physical activity, which further exacerbates the decrease in cardiorespiratory fitness and thus the decrease in executive function. Increased SSB consumption is often associated with decreased cardiorespiratory fitness in a vicious cycle, further exacerbating SSB consumption or decreased cardiorespiratory fitness levels (50). Therefore, this result suggests that SSB consumption in adolescents at high altitudes should be effectively controlled in the future and that cardiorespiratory fitness levels should be effectively increased to better promote adolescent executive function levels.

The present study has certain strengths and limitations. Strengths: First, to the best of our knowledge, this study analyzed for the first time the association of SSB consumption and cardiorespiratory fitness with executive function in Tajik adolescents in high-altitude areas of Xinjiang, China. This study accumulates basic information for enriching the research related to executive function in adolescents in high-altitude areas of China. Second, the geographic area of this study is high altitude and the participants are Tajik adolescents, which is typical of the geographic area and provides research information for the study of the executive function of ethnic minority groups. However, there are some limitations to this study. First, the age group investigated in this study was adolescents in the 13–15 years age group, which is at the peak of pubertal development, which may have an impact on the associations between SSB consumption and cardiorespiratory fitness with executive function due to pubertal development. In addition, the findings of the 13–15 year olds in this study may not be applicable to adolescents in other age groups during puberty, and more age groups should be included in future studies. Second, the present study was a cross-sectional study, which could only analyze the associations between SSB consumption and cardiorespiratory fitness with executive function in adolescents, but not the causal associations. Future prospective cohort studies should be conducted to analyze the existence of causal associations. Third, the assessment of cardiorespiratory fitness in this study was conducted indirectly by using the 1,000-meter run for boys and the 800-meter run for girls, which may have some deviation from the actual cardiorespiratory fitness of adolescents. In the future, more objective measurement instruments, such as a running platform combined with a gas analyzer, should be used for objective assessment to accurately reflect the correlation between cardiorespiratory fitness. Fourth, the absence of a baseline investigation of heart rate, blood pressure, respiratory rate, and oxygen saturation in Chinese Tajik adolescents is also one of the limitations of this study. These research indicators should be included in the future to improve the comprehensiveness of the study. Sixth, SSB consumption was only assessed in this study over the past month for participants, which does not capture long-term trends in SSB consumption and health outcomes and is a shortcoming of this study. Future assessments should be conducted over a longer period of time to provide more reliable data on SSB consumption. Finally, the covariates included in this study were limited, and factors that are important in influencing executive function, such as socioeconomic status, daily caloric intake, and place of residence, should be included in the future to further strengthen the analysis.

## 5 Conclusion

SSB consumption and cardiorespiratory fitness were associated with executive function in Tajik adolescents from Xinjiang, a high-altitude region of China. Increased SSB consumption and decreased cardiorespiratory fitness increased the risk of executive function disorders in Tajik adolescents. In the future, SSB consumption and cardiorespiratory fitness should be effectively controlled to improve the executive function of Tajik adolescents and to promote the physical and mental health of Tajik adolescents in high-altitude areas.

## Data Availability

The raw data supporting the conclusions of this article will be made available by the authors, without undue reservation.
